# Lysine-Specific Demethylase 1 (LSD1/KDM1A) Contributes to Colorectal Tumorigenesis via Activation of the Wnt/Β-Catenin Pathway by Down-Regulating Dickkopf-1 (DKK1)

**DOI:** 10.1371/journal.pone.0070077

**Published:** 2013-07-26

**Authors:** Zebin Huang, Shangze Li, Wei Song, Xin Li, Qinshan Li, Zeyan Zhang, Yongqing Han, Xiaodong Zhang, Shiying Miao, Runlei Du, Linfang Wang

**Affiliations:** 1 National Laboratory of Medical Molecular Biology, Department of Biochemistry and Molecular Biology, Institute of Basic Medical Sciences, Chinese Academy of Medical Sciences, Peking Union Medical College, Tsinghua University, Beijing, China; 2 Department of Cellular and Developmental Biology, College of Life Sciences, Wuhan University, Wuhan, China; University of Alabama at Birmingham, United States of America

## Abstract

We collected paired samples of tumor and adjacent normal colorectal tissues from 22 patients with colorectal carcinoma to compare the differences in the expression of lysine specific demethylase 1 (LSD1) in these two tissues. The results showed that in 19 paired samples (86.4%), LSD1 is more highly expressed in tumor tissue than in normal tissue. To explore the role of LSD1 in colorectal tumorigenesis, we used somatic cell gene targeting to generate an LSD1 knockout (KO) HCT 116 human colorectal cancer cell line as a research model. The analysis of phenotypic changes showed that LSD1 KO colorectal cancer cells are less tumorigenic, both in vivo and in vitro. The differential expression analysis of the cells by mRNA sequencing (RNA-Seq) yielded 2,663 differentially expressed genes, and 28 of these genes had highly significant differences (Q <0.01). We then selected the 4 colorectal cancer-related genes ADM, DKK1, HAS3 and SMURF2 for quantitative real-time PCR verification. The results showed that the differences in the expression of ADM, DKK1 and HAS3 were consistent with those measured using the RNA-Seq data. As DKK1 was the gene with the most significant differential expression, we analyzed the key proteins of the DKK1-related Wnt/β-catenin signaling pathway and found that, after knocking out LSD1, the amount of free β-catenin translocated to the nucleus was significantly reduced and that the transcription of the signaling pathway target gene c-Myc was down-regulated. Our studies show that LSD1 activates the Wnt/β-catenin signaling pathway by down-regulating the pathway antagonist DKK1, which may be one of the mechanisms leading to colorectal tumorigenesis.

## Introduction

Colorectal cancer is one of the most prevalent types of malignant cancer worldwide. As in other cancers, the development of colorectal cancer requires the involvement of multiple genes and the gradual accumulation of mutations, which is primarily related to the instability of oncogenes, cancer suppressor genes, and growth factors [1]. A detailed understanding of the molecular mechanisms and signaling pathways may provide knowledge for the treatment and prevention of colorectal cancer.

Histone methylation is a major determinant of chromatin structure and function and has been shown to play critical roles in heterochromatin formation, X-chromosome inactivation, transcriptional regulation and DNA repair [2]. Histone methylation was considered to be a permanent epigenetic mark until the first demethylase, named lysine specific demethylase 1 (LSD1, also known as KDM1A and AOF2), was identified in 2004 [3]. Since then, histone methylation has been shown to be a dynamic process that is regulated via the addition of methyl groups by methylases and the removal of methyl groups by demethylases. As a member of the monoamine oxidase (MAO) family, LSD1 catalyzes the specific demethylation of both mono- and di-methylated lysine 4 (K4) or lysine 9 (K9) of histone H3 (H3K4me1/2 or H3K9me1/2) via a process that requires flavin adenine dinucleotide (FAD) as an essential redox cofactor [3], [4]. The methylation of specific lysine residues on histones has been linked with either transcriptional activation or repression: H3K4 methylation correlates with transcriptional activation, while H3K9 methylation with repression [5]. Thus, LSD1 plays an important role in transcriptional regulation [3], [6]. It can selectively initiate or repress the transcription of target genes via demethylation of H3K4 or H3K9 by interacting with a variety of molecular partners. LSD1 participates in transcriptional repression as part of various protein complexes that contain several transcriptional corepressors, including the REST, CoREST, RCOR2, BHC80, HDAC1/2, CtBP, BRAF35, NuRD etc. [7], [8], [9], [10], and mediates the demethylation of H3K4me1/2. In contrast, when LSD1 interacts with androgen receptor (AR) or estrogen receptor (ER), it mediates the demethylation of H3K9me1/2 to promote transcription [3], [4]. In addition to histone substrates, LSD1 has also been shown to demethylate lysine residues at several non-histone substrates, such as p53 [11], Dnmt1 [12], E2F1 [13], and MYPT1 [14]. Additional substrates of LSD1 remain to be discovered. The ability to regulate such a wide range of substrates has linked LSD1 to a broad spectrum of biological processes, including cell proliferation [15], chromosome segregation [16], hematopoiesis [17], spermatogenesis [18], adipogenesis [19], regulation of stem cell pluripotency [20], and embryonic development [21]. The dysregulation of LSD1 activity has an important role in human tumorigenesis [14], and studies have already been performed on breast cancer [22], prostate cancer [23], leukemia [24], lung cancer [25], bladder cancer [26], neuroblastoma [27], and colorectal cancer [28], etc. However, the role of LSD1 in colorectal cancer has not been elucidated.

Dickkopf-1 (DKK1), a secreted protein, is known to be a negative regulator of the Wnt/β-catenin signaling pathway. This pathway plays an important role in development and in regulating adult stem cell systems. A variety of cellular processes are mediated by Wnt/β-catenin signaling, including proliferation, differentiation, survival, apoptosis, and cell motility [29]. Dysregulation of the pathway leads to tumorigenesis in several types of human cancers, including colorectal cancer [30], [31], [32], [33], [34].

Both LSD1 and DKK1 are associated with tumorigenesis. However, to the best of our knowledge, there is no report discusses these two proteins simultaneously. Here, we show for the first time that LSD1 contributes to colorectal tumorigenesis by down-regulating DKK1.

## Materials and Methods

### Ethics Statement

Signed informed consent was obtained from all patients for sample collection and analysis. The study was conducted in accordance with the Helsinki Declaration for human subjects studies and was approved by the Institutional Review Board of the Cancer Hospital, Chinese Academy of Medical Sciences (CAMS), Beijing, China. All animal experiments were performed in strict accordance with the recommendations in the Regulations for the Administration of Affairs Concerning Experimental Animals as Established by the State Science and Technology Commission of the People’s Republic of China. The protocol was approved by the Committee on the Ethics of the Care and Use of Laboratory Animals of Wuhan University (Permit Number: 11100e). All surgery was performed under sodium pentobarbital anesthesia, and all efforts were made to minimize suffering.

### Clinical Specimens

Paired samples of tumor and adjacent normal colorectal tissues were obtained at the time of surgery from 22 patients with colorectal carcinoma at Cancer Hospital, CAMS, Beijing, China. Immediately after surgical resection, the specimens were snap-frozen in liquid nitrogen and stored at −80°C until use.

### Cell Lines and Culture

DLD-1, HCT 116, HT29, RKO, SW480, CT26 and HEK 293T cells were purchased from American Type Culture Collection (ATCC). DLD-1, and CT26 were cultured in RPMI 1640 medium supplemented with 10% fetal bovine serum (FBS); HCT 116 and HT29 were cultured in McCoy’s 5a medium supplemented with 10% FBS; RKO was cultured in Eagle’s Minimum Essential medium supplemented with 10% FBS; SW480 was cultured in Leibovitz’s L-15 medium supplemented with 10% FBS; HEK 293T was cultured in Dulbecco’s Modified Eagle’s medium supplemented with 10% FBS. All cell lines were cultured in a humidified atmosphere containing 5% carbon dioxide (CO_2_) and 95% air at 37°C. All of the media and FBS were purchased from HyClone.

### Somatic Cell Gene Targeting

Somatic cell gene targeting was performed as described previously [35], [36]. Briefly, an 837 bp fragment from intron 6 to exon 7 of the LSD1 locus was amplified for 25 cycles from genomic DNA as the left homology arm (HA) using the following primers: P1, 5′-GGGAAAGUGGACCTCCTACCTGGCTGAT-3′ and P2, 5′-GGAGACAUTCA TTGTAACTGTCGAGCTGCTG-3′. Another 1041 bp fragment from exon 7 to intron 7 of the LSD1 locus was also amplified for 25 cycles from genomic DNA as the right HA using the following primers: P3, 5′-GGTCCCAUTTGGAAGCCAGGGTAAGAA-3′ and P4, 5′-GGCATAGUCTTACACCCTGATCCCCAAA-3′. Both the left and right HAs were cloned into a pAAV-LoxP-Neo knockout (KO) vector with USER enzyme (New England Biolabs). The targeting adeno-associated viruses (AAV) were packaged in 293T cells (one well of a 6-well plate at 60% confluence) by transfecting equal amounts of the targeting vector, pAAV-RC and pAAV-Helper plasmids (3 µg each). Viruses were harvested at 72 hours post-transfection. HCT 116 cells were infected with the LSD1 KO targeting viruses and selected with geneticin (G418) for 2 weeks. The G418 resistant clones were then screened for homologous recombination by 35 cycles of genomic PCR with primers derived from the upstream region of the left HA (P5, 5′-TGTCCACCGAGTTCACAGTT-3′) and the neomycin resistance gene (P6, 5′-GGGGTTTGCTCGACATTG-3′). Confirmatory genomic PCR was also carried out with positive clones identified by primers derived from the neomycin resistant gene (P7, 5′-TCGCCTTCTTGACGAGTTCT-3′) and the downstream region of the right HA (P8, 5′-CAGGGCCACCCATATAGAAA-3′). To target the second allele with the same targeting viruses, the correctly targeted clones were infected with adenoviruses expressing the Cre-recombinase to delete the drug selection marker. The clones with successful deletion of the drug selection marker were selected by performing 30 cycles of genomic PCR, which was performed to amplify an approximately 300-bp genomic fragment in which the LoxP site was inserted (using primers P9, 5′-GCTTGGCAGCAGCTCGACAGTTA-3′ and P10, 5′-AACCCTCTTCACAGAGTACTTTTC-3′). The heterozygous KO clones were infected with the same targeting viruses to target the second allele, and the neomycin resistance gene was excised as described earlier.

### Re-expression of LSD1 in KO Cell

Full-length human LSD1 cDNA, amplified from HCT 116 cells by reverse transcription PCR (RT-PCR), was subsequently cloned into the Eukaryotic Expression Vector pcDNA3.1(+) (Invitrogen), and recombinant plasmid of pcDNA-LSD1 was constructed. Subsequently, the pcDNA-LSD1 plasmid was transfected into LSD1 KO HCT 116 cells, and selected with G418. The re-expression of LSD1 in LSD1 KO HCT 116 cell was determined by Western blot analysis.

### Western Blot Analysis

Total protein extracts from cells and the tumor and corresponding adjacent normal colorectal tissues were prepared in lysis buffer (Promega) containing the complete cocktail of protease inhibitors (Roche). Nuclear and cytoplasmic proteins were separated using the NE-PER nuclear and cytoplasmic extraction kit (Pierce) following the manufacturer’s instructions. The protein concentrations were determined with the BCA protein assay reagent (Pierce). Proteins were separated by SDS-PAGE and then transferred to polyvinylidene fluoride (PVDF) membranes. After blocking, the membranes were incubated with specific primary antibodies. After three washes, the membranes were further incubated with appropriate horseradish peroxidase-conjugated secondary antibodies (Golden Bridge). Specific protein bands were developed using a chemiluminescence-based system ECL. The intensity of each band was quantified using AlphaEase®FC software after densitometric scanning of the films. The following primary antibodies were used: LSD1 (AP1218c) from ABGENT; H3K4me1 (07-436), H3K4me2 (07-030), H3K9me1 (07-450) and H3K9me2 (07-212) from Millipore; H3 (ab1791) from abcam; β-catenin (9587), Phospho-β-catenin (9561), c-Myc (5625P) and CREB (9197) from Cell Signaling Technology; DKK1 (Sc-25516) from Santa Cruz; β-actin (A1978) and GAPDH (G8795) from Sigma.

### Proliferation Assay

Approximately 1,000 cells per well were seeded in 96-well plates and cultured for 1, 2, 3, 4, 5, and 6 days, respectively. Cell Counting Kit-8 (CCK-8) (DOJINDO) solution was added to each well and incubated for 1 hour at 37°C, and the absorbance at 450 nm was measured using a microplate reader. Each assay was performed on five replicate samples and repeated three times.

### Plate Colony Formation Assay

Approximately 200 cells per well were seeded in 6-well plates. The cells were cultured for more than 10 days until visible colonies were formed and were then stained with crystal violet, photographed, and the colonies were counted. Each assay was performed in triplicate and repeated three times.

### Soft Agar Colony Formation Assay

Approximately 1.0×10^4^ cells per well were suspended in 0.35% soft agar on top of a 0.7% soft agar base layer in 6-well plates; each layer consisted of 2 mL soft agar containing complete growth medium. The cells were cultured for more than 10 days until visible colonies were formed and then the colonies were photographed and counted. Each assay was performed in triplicate and repeated three times.

### Xenograft Experiments

Six-week-old athymic nude mice were purchased from HFK. Cells from each stable line were concentrated to 2.0×10^6^ per 200 µL and injected subcutaneously (s.c.) into both lower flanks of the mice (6 mice in each group). The mice were kept under pathogen-free conditions and fed with a standard diet. Beginning 6 days later, the tumor diameters were measured using a caliper every 3 days, and the approximate tumor volume in mm^3^ was calculated by the following formula: volume = (a×b^2^)/2, where a is the long axis and b is the short axis of the tumor. On the 22^nd^ day, the mice were sacrificed, and the tumors were explanted and weighed.

### RNA Sequencing (RNA-Seq)

Cell samples were used for single-end mRNA sequencing performed by EMTD Technology Development Co. Ltd. using a HiSeq2000 instrument (illumina). To compare mRNA expression data before and after LSD1 KO, read counts for each identified mRNA were normalized to the total number of reads in each given sample. Differential expression was evaluated using the fragments per kilobase of transcript per million fragments mapped (FPKM) method. The fold change of mRNA reads was calculated as the ratio of the KO group to the wild-type (WT) group, and Q values <0.01 were considered to indicate highly significant differences.

### Quantitative Real-time PCR (qPCR)

Total cellular RNA was extracted using TRIZOL reagent (Invitrogen). Reverse transcription was performed using the ReverTra Ace -α- kit (TOYOBO). qPCR was carried out in triplicate using the Power SYBR Green Master Mix (Applied Biosystems) with a StepOnePlus (Applied Biosystems) sequence detection system. The cycling conditions were 95°C for 10 min, followed by 40 cycles of 95°C for 15 sec and 60°C for 60 sec. Amplification of β-actin was performed in parallel for each sample and used as an internal control. The following primer pairs were used: 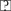 for HAS3: forward, 5′-CGCAGCAACTTCCATGAGG-3′ and reverse, 5′-AGTCGCACACCTGGATGTAGT-3′; 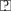 for ADM: forward, 5′-GACACCGCTCGGTTGGATG-3′ and reverse, 5′-CAGAGCCCACTTATTCCACTTC-3′; 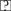 for SMURF2: forward, 5′-TATGCAAACTCGGGCCAAATG-3′ and reverse, 5′-CCTGTGCCTATTCGGTCTCTG-3′; 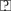 for DKK1: forward, 5′-ATAGCACCTTGGATGGGTATTCC-3′ and reverse, 5′-CTGATGACCGGAGACAAACAG-3′.

### Enzyme-linked Immunosorbent Assay (ELISA)

DKK1 levels in supernatants from cultured cells were measured using a commercial DKK1 ELISA Kit (Jiamay). Prior to the assay, approximately 1.0×10^6^ cells per well were seeded in 6-well plates, and the culture media were harvested after 48 hours of culture. The ELISA was performed following the manufacturer’s instructions. Data were expressed as concentration in ng/mL produced by 1.0×10^6^ cells in 48 hours. Each assay was performed in triplicate and repeated three times.

### Statistical Analysis

All experiments were independently performed at least three times in triplicate. Statistical analysis was performed using GraphPad Prism 5 software. P values were calculated using Student’s t-test. P values <0.05 were considered significant, while P values <0.01 were considered highly significant. The data are presented as the mean ± SD.

## Results

### LSD1 is Highly Expressed in Tumor Colorectal Tissues in Comparison to Adjacent Normal Colorectal Tissues

**Figure 1:**
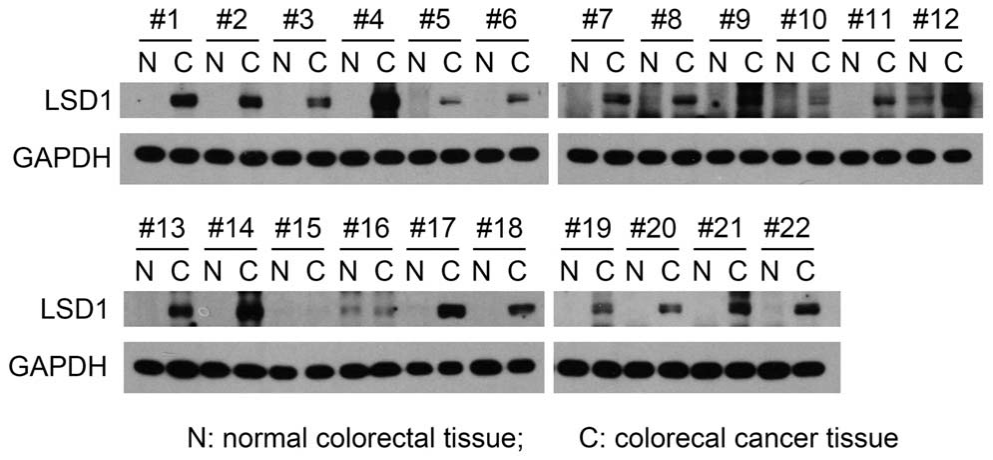
Expression differences of LSD1 between tumor and adjacent normal colorectal tissues. GAPDH was used as an internal control. #1∼#22, paired tissue samples 1∼22.

We collected paired samples of tumor and adjacent normal colorectal tissues from 22 patients with colorectal carcinoma to compare the differences in the expression of LSD1 in the two tissues. The result showed that in 19 pairs (86.4%) of samples, LSD1 was highly expressed in tumor tissues compared to adjacent normal tissues (Figure 1). This result suggested that LSD1 might function in colorectal tumorigenesis.

### Generation of LSD1 KO HCT 116 Human Colorectal Cancer Cell Line as a Research Model for Colorectal Cancer

**Figure 2:**
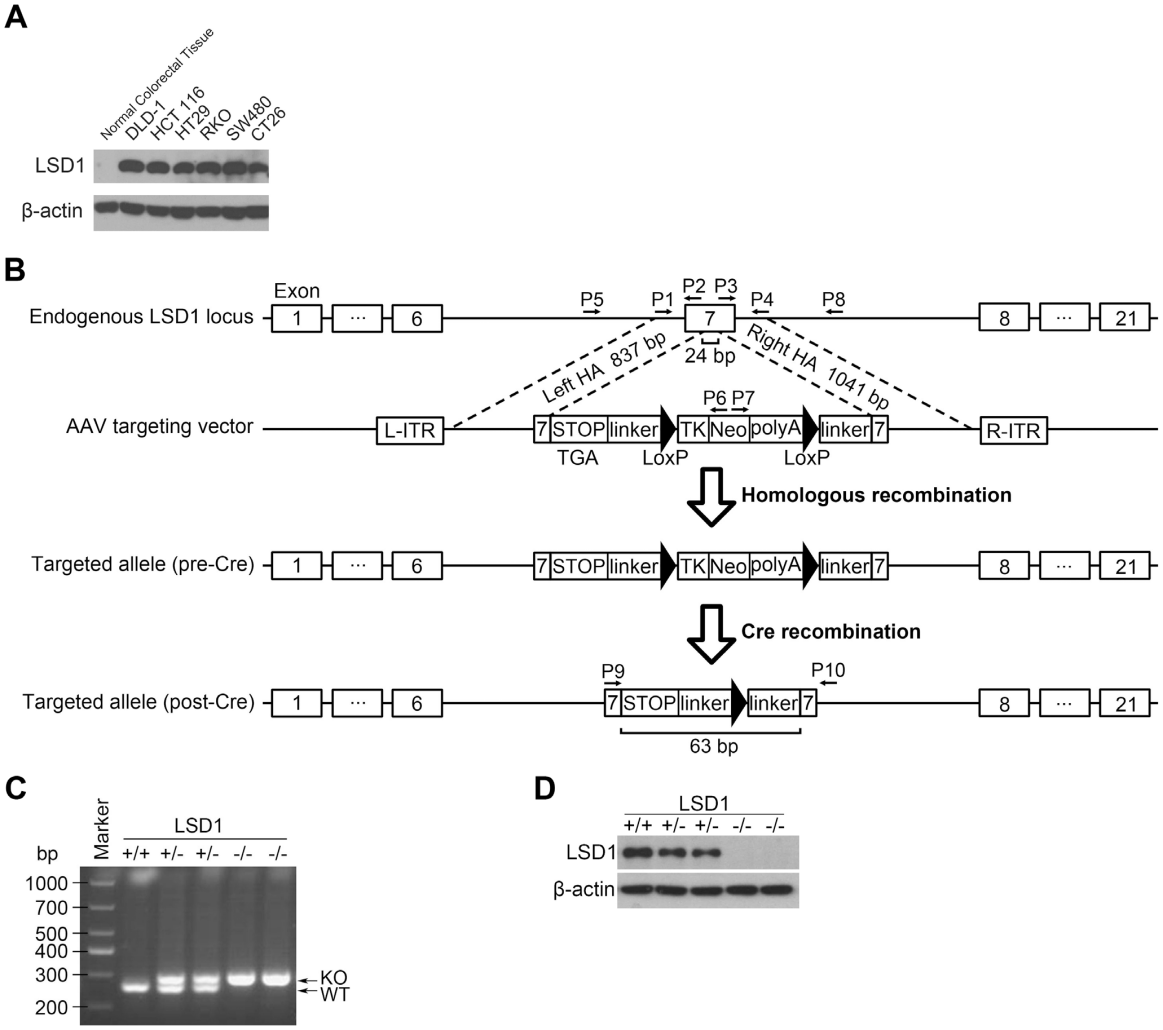
Generation of the LSD1 KO colorectal cancer cell line. (A) Selection of colorectal cancer cell lines. (B) Diagram of the targeting strategy. The endogenous LSD1 locus is shown, along with the AAV targeting vector and the targeted allele before and after Cre-mediated recombination. PCR primers P1∼P10 were described in Materials and Methods. Three STOP codons TGA were added at the end of the left HA to ensure premature termination of the transcript. L-ITR and R-ITR, left and right inverted terminal repeats, respectively; HA, homology arm; TK, TK promoter; Neo, neomycin resistance gene; polyA, polyadenylation signal. (C) Identification of LSD1 null cell lines by genomic PCR. PCR detected the WT and targeted alleles from the indicated cells using genomic DNA as template. Two independent clones for the KO are shown. The lower row with the band designated “WT” indicates that the lower band was amplified from wild-type allele. The upper row with the band designated “KO” shows that the upper band was amplified from the allele with part of exon 7 of the LSD1 knocked out and LoxP inserted. (D) Confirmation of LSD1 null cell lines by Western blot analysis. β-actin was used as an internal control.

To explore the role of LSD1 in colorectal tumorigenesis, we used somatic cell gene targeting to generate a LSD1 KO colorectal cancer cell line as a colorectal cancer research model. We first analyzed LSD1 expression in various colorectal cell lines, including DLD-1, HCT 116, HT29, RKO, SW480 and CT26, to find one in which LSD1 was highly expressed and suited for use as the model. The result showed that all of the cell lines highly expressed LSD1 (Figure 2A). Because HCT 116 had been widely used for successful gene targeting by homologous recombination [35], [37], we chose HCT 116 to generate the LSD1 KO research model. The targeting strategy is outlined in the schematic diagram in Figure 2B. Recombinant adeno-associated virus (rAAV) vectors provide an efficient means of permanently inactivating genes in human cancer cells [38], [39]. In this work, we generated targeting vectors with a neomycin resistance gene so that infected cells would express the drug resistance gene only when homologous recombination occurs. Targeting was directed to exon 7 of the LSD1 gene (Figure 2B). Targeting was performed in a colon cancer cell line, HCT 116, and was initially evaluated via PCR of genomic DNA. Following disruption of the first allele, Cre recombinase was used to remove the drug-resistance cassette integrated at the targeted locus, permitting another round of targeting to disrupt the second allele. This process of targeting by rAAV infection followed by Cre recombinase-mediated excision was performed twice in sequence to disrupt both alleles of LSD1. PCR of genomic DNA from each clone was performed to verify the nature of the targeting events (Figure 2C). Finally, Western blot analysis was used to confirm the absence of protein expression (Figure 2D). Both PCR and Western blot analysis confirmed the heterozygous (LSD1^+/−^) and homozygous (LSD1^−/−^) gene disruption, and we obtained two independent LSD1 KO heterozygous and homozygous clones for these studies.

### LSD1 KO Colorectal Cancer Cells Grew Slower than WT Cells

**Figure 3:**
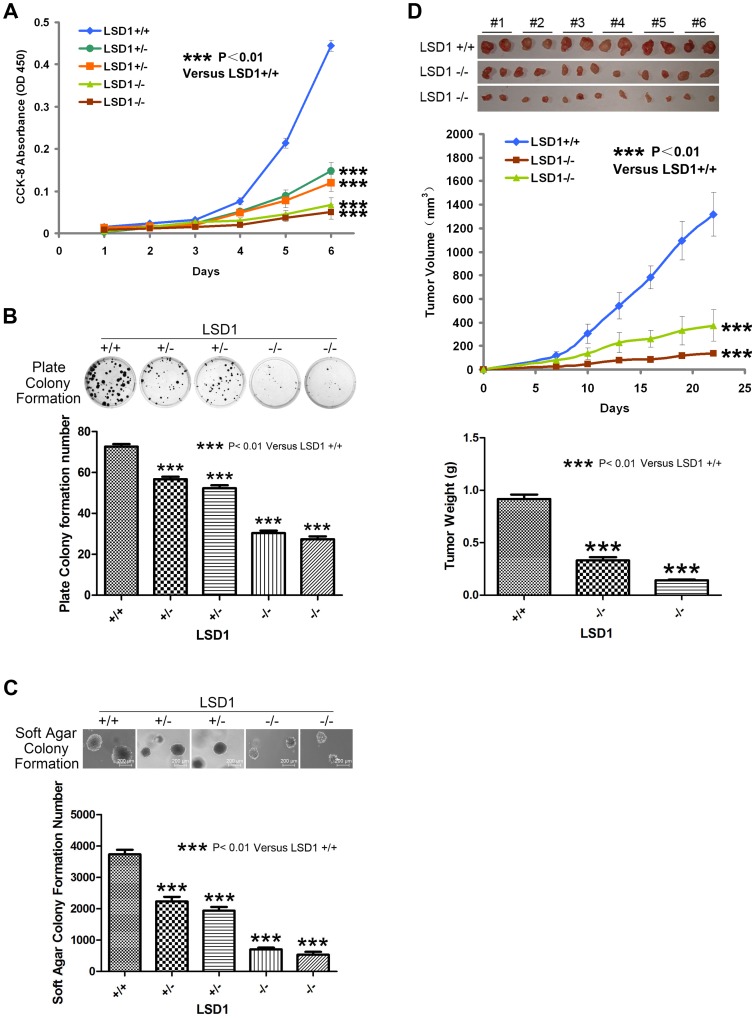
LSD1 KO colorectal cancer cells are less tumorigenic both in vivo and in vitro. (A) Proliferation assays. Approximately 1,000 cells per well of the indicated cell lines were seeded in 96-well plates. The proliferation of each clone was measured daily for 6 consecutive days by the CCK-8 method (measuring the absorbance at 450 nm) and plotted. (B) Plate colony formation. Approximately 200 cells per well of the indicated cell lines were seeded in 6-well plates. After 10 days in culture, the cells were stained with crystal violet, the colonies were counted and the number of colonies was plotted for each of the clones. The experiments were performed in triplicate; representative wells are shown. (C) Soft agar colony formation. Approximately 1.0×10^4^ cells per well of the indicated cell lines were suspended in soft agar medium in 6-well plates. After 10 days in culture, the colonies were then counted, and the number of colonies was plotted for each of the clones. The experiments were performed in triplicate; representative wells are shown. (D) Xenograft experiments. LSD1 KO colorectal cancer cells are less tumorigenic in vivo. Athymic nude mice were injected subcutaneously with cells from the indicated clones. Tumor sizes were measured every 3 days for approximately 3 weeks and plotted. The mice were then sacrificed, and the tumors were harvested and weighed. Each mouse grew two tumors. #1∼#6, mouse 1∼6 in each group.

CCK-8 allows sensitive colorimetric assays for the determination of the number of viable cells in cell proliferation assays. WST-8, used in CCK-8, is reduced by dehydrogenase in the cells to give an orange colored product (formazan), which is soluble in the culture medium. The amount of the formazan dye generated by dehydrogenase in cells is directly proportional to the number of living cells. To test whether LSD1 KO affected proliferation, growth was measured by CCK-8, and the cell growth curve was plotted. The result showed that the homozygous KO cells grew much more slowly than WT cells and that the growth rate of the heterozygous KO cells was between those of the two cell lines mentioned above (Figure 3A).

### In vitro, LSD1 KO Colorectal Cancer Cells Exhibited Reduced Colony-forming Abilities

To test whether LSD1 KO affected tumorigenicity-correlated responses in vitro, we performed plate colony formation and soft agar colony formation assays. Compared with the WT cells, the homozygous KO cells exhibited a highly significant (P<0.01) reduced colony-forming ability in the plate colony formation assay and the soft agar formation assay. Notably, the colony-forming ability of the heterozygous KO cells was between those of the two cell lines mentioned above (Figure 3B and C).

### LSD1 KO Colorectal Cancer Cells were Less Tumorigenic in vivo

Tumorigenicity of the KO cells was also tested in an in vivo model. For these studies, LSD1 KO homozygous clones or WT HCT 116 cells were injected subcutaneously into nude mice. Beginning 7 days later, tumor sizes were assessed every 3 days by caliper measurements. The homozygous KO cells produced tumors that grew more slowly than those produced by the WT cells (P<0.01). After 22 days of growth, the average tumor volume of the homozygous KO cells was approximately 5-fold smaller than those produced by the WT cells (P<0.01) (Figure 3D).

### Re-expression of LSD1 in the KO Cell Restored the Phenotype

**Figure 4:**
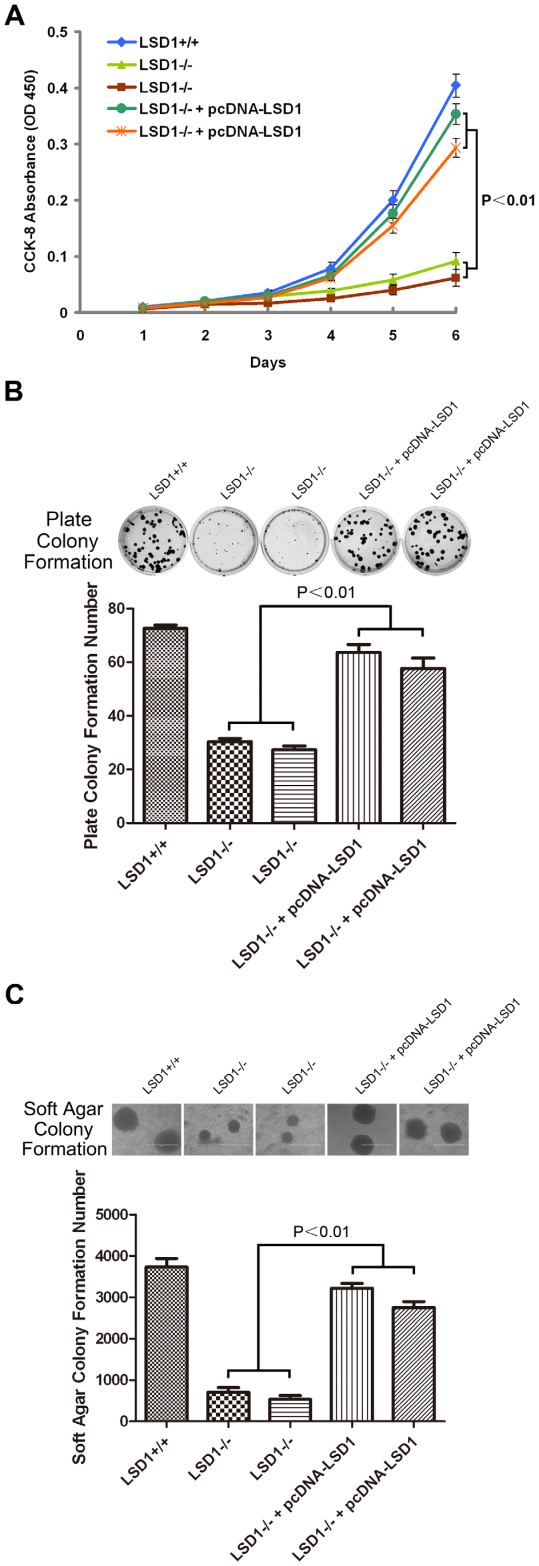
Re-expression of LSD1 in the KO cell restored the phenotype. (A) Proliferation assays. (B) Plate colony formation. (C) Soft agar colony formation.

To verify that the observed phenotype was due to disruption of the LSD1 gene and not simply due to some other mutation in the cell line, we re-expressed LSD1 in the KO cell and then repeated the cell proliferation assays and colony formation assays. Notably, the re-expression of LSD1 was sufficient to restore the phenotype (Figure 4A, B and C).

### LSD1 KO did not Increase Global Histone Methylation

**Figure 5:**
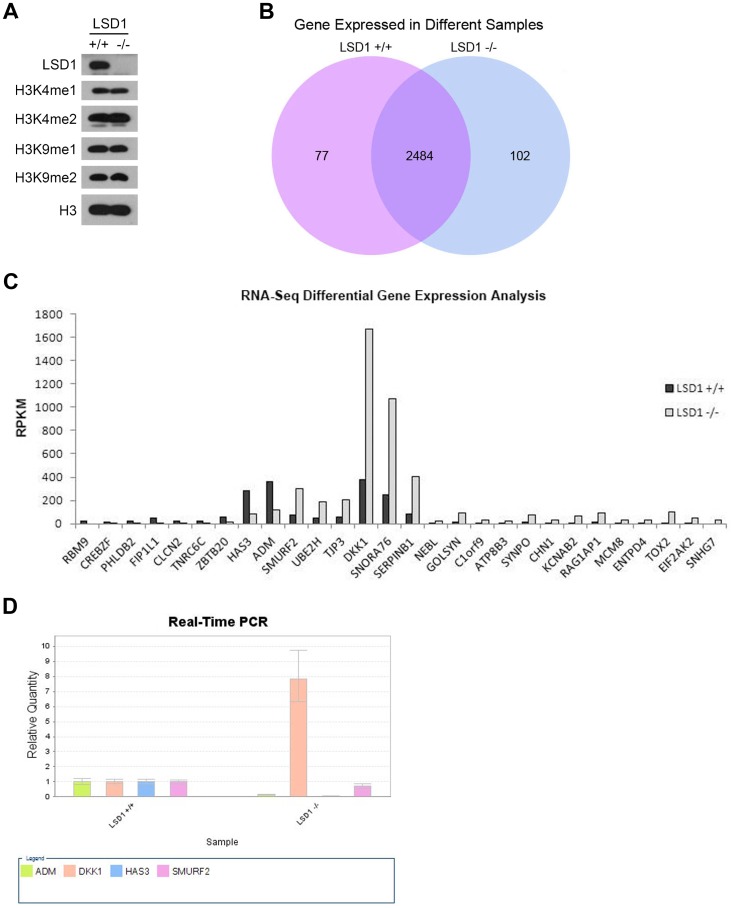
Global histone methylation and gene expression in WT and KO cell lines. (A) Western blot analysis of the global methylation levels of H3K4me1/2 and H3K9me1/2 of the cells before and after knocking out LSD1. H3 was used as an internal control. (B) Gene expression in WT and KO cell lines by RNA-Seq analysis. (C) RNA-Seq differential gene expression analysis of the cells before and after knocking out LSD1. Q values <0.01. (D) qPCR verification of gene expression in WT and KO cell lines. Validation of the RNA-Seq data using the qPCR assay to establish expression ratios between the LSD1 KO and WT cell lines. The total RNA used for the qPCR assay was obtained one set of the three replicate experiments, and the qPCR data shown represent the average of three independent reactions.

LSD1 is highly expressed in tumor colorectal tissues compared to adjacent normal colorectal tissues, and LSD1 KO colorectal cancer cells are less tumorigenic both in vivo and in vitro, which suggested that LSD1 functions in colorectal tumorigenesis. To explore the molecular basis of LSD1 and tumorigenesis, a series of experiments was performed in this laboratory. LSD1 is a demethylase, and it catalyzes the specific demethylation of H3K4me1/2 or H3K9me1/2. Here, we used Western blotting to detect changes in the global methylation levels of H3K4me1/2 and H3K9me1/2 in the cells before and after knocking out LSD1. The results showed that these global methylation levels did not change significantly (Figure 5A).

### Transcriptional Effects of LSD1 KO

RNA-Seq was performed to analyze the differential expression of the cells before and after knocking out LSD1. Here, we found 2,663 differentially expressed genes; 102 of these genes were only expressed in the homozygous KO cells, whereas 77 were only expressed in WT cells (Figure 5B and Table S1). Statistical analysis allows us to screen out 28 genes that exhibited highly significant differences (Q <0.01). Of these 28 genes, 9 were down-regulated after knocking out LSD1, and the remaining 19 were up-regulated (Table 1 and Figure 5C).

**Table 1:** RNA-Seq differential gene expression analysis of the cells before and after knocking out LSD1. Q values <0.01.

	Chromosome	Gene Name	Accession Num	GI	RPKM LSD1+/+	RPKM LSD1−/−	RPKM Ratio
**Down-regulated genes**	chr22∶36134782-36425014	RBM9	NM_001082578	133925802	22.3567	0	
	chr11∶85368607-85376182	CREBZF	NR_028026	253735743	17.6371	0.920806	19.15
	chr3∶111393512-111695364	PHLDB2	NM_001134438	197313729	25.4097	2.82168	9.01
	chr4∶54243763-54326181	FIP1L1	NM_030917	201023337	46.0611	5.4131	8.51
	chr3∶184062279-184079936	CLCN2	NM_004366	283806614	18.5061	2.21267	8.36
	chr17∶76000258-76105687	TNRC6C	NM_001142640	217416331	17.9741	3.53404	5.09
	chr3∶114056911-114790261	ZBTB20	NM_001164343	257900534	60.0398	13.33	4.50
	chr16∶69140054-69151595	HAS3	NM_005329	20302152	282.114	82.9034	3.40
	chr11∶10326517-10328943	ADM	NM_001124	4501944	358.132	113.56	3.15
**Up-regulated genes**	chr7∶129470574-129592817	UBE2H	NM_003344	321267497	48.7407	185.244	0.26
	chr17∶62539672-62658471	SMURF2	NM_022739	56550041	77.4254	297.909	0.26
	chr19∶3728373-3751548	TJP3	NM_014428	10092690	52.9945	208.328	0.25
	chr17∶62223242-62340653	SNORA76	NR_002995	91754179	251.869	1076.79	0.23
	chr10∶54074000-54077417	DKK1	NM_012242	61676924	382.42	1673.1	0.23
	chr6∶2833078-2842264	SERPINB1	NM_030666	20149554	87.1642	407.857	0.21
	chr10∶21068901-21186531	NEBL	NM_006393	197313771	5.62632	26.5163	0.21
	chr8∶110586404-110657822	GOLSYN	NM_001099750	153090196	16.7722	89.617	0.19
	chr19∶1782073-1812270	ATP8B3	NM_138813	55743078	3.96713	21.6199	0.18
	chr1∶172502121-172580973	C1orf9	NM_014283	170784827	5.49381	30.9739	0.18
	chr5∶149980641-150038792	SYNPO	NM_007286	261278294	9.80218	70.6967	0.14
	chr2∶175664041-175870170	CHN1	NM_001822	331028574	4.47354	32.9868	0.14
	chr1∶6086342-6161240	KCNAB2	NM_172130	315434243	7.08796	63.0558	0.11
	chr1∶155108287-155111334	RAG1AP1	NM_018845	170932468	9.50747	95.1037	0.10
	chr20∶5931297-5976791	MCM8	NM_182802	33469923	2.76332	32.6384	0.08
	chr8∶23286596-23315441	ENTPD4	NM_004901	347543761	2.02587	29.3349	0.07
	chr2∶37331356-37384319	EIF2AK2	NM_001135651	351542234	2.91037	46.3784	0.06
	chr20∶42543436-42698256	TOX2	NM_032883	149408142	5.61672	99.022	0.06
	chr9∶139614545-139622636	SNHG7	NR_024543	215422348	0	29.3914	0.00

### Validation of mRNA Sequencing Data of 4 Colorectal Cancer-related Genes

We selected 4 colorectal cancer-related genes, ADM, DKK1, HAS3 and SMURF2, from the 28 genes mentioned above for further confirmation using qPCR. The total RNA used for the qPCR experiments was obtained one set of the three replicate experiments, and the qPCR data shown represent the average of three independent reactions. The result showed that the differences in expression of ADM, DKK1 and HAS3 were consistent with those measured using the RNA-Seq data: ADM and HAS3 were down-regulated, whereas DKK1 was up-regulated after knocking out LSD1. However, SMURF2 expression was not significantly different before and after knocking out LSD1, as shown by qPCR (Figure 5D).

### LSD1 Activated the Wnt/β-catenin Signaling Pathway by Down-regulating the Pathway Antagonist DKK1

**Figure 6:**
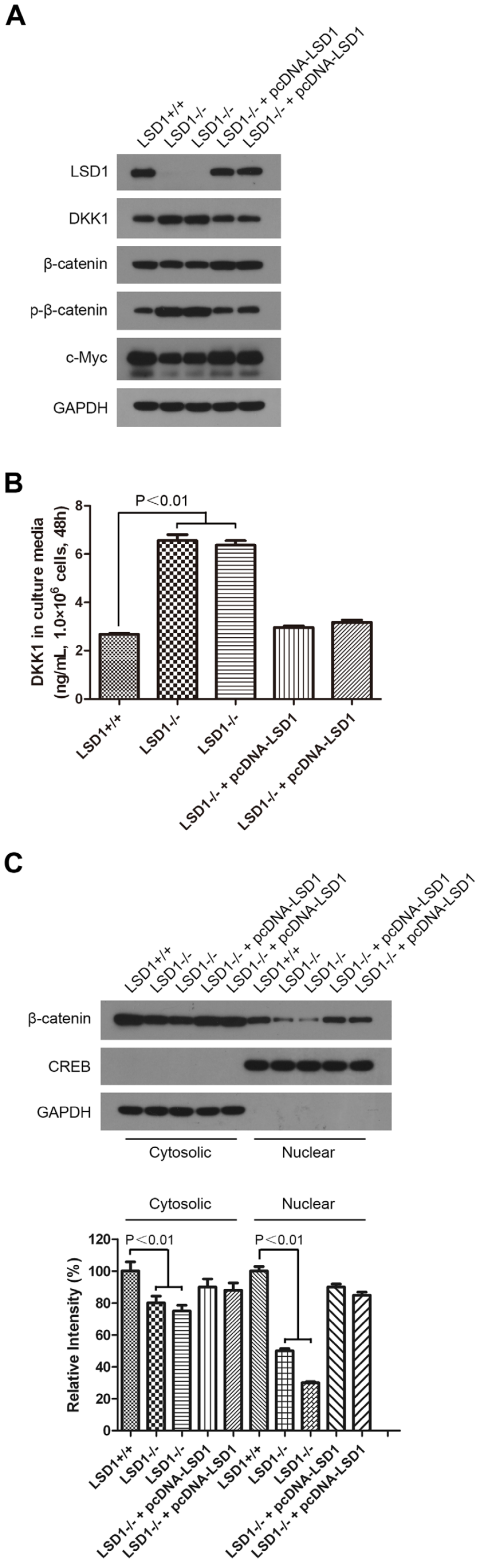
Mechanisms of LSD1-induced colorectal tumorigenesis mediated by down-regulating DKK1. (A) Western blot analysis of key proteins of the Wnt/β-catenin pathway in WT and KO cell lines. GAPDH was used as an internal control. (B) ELISA analysis of DKK1 in culture media of WT and KO cell lines. (C) Western blot analysis of β-catenin in the cytoplasm and the nucleus of WT and KO cell lines. GAPDH was used as an internal control for the cytosolic proteins, and CREB was used as an internal control for the nuclear proteins. The intensity of each band was quantified using AlphaEase®FC software after densitometric scanning of the films.

Because DKK1 had the most significant differential expression before and after LSD1 KO in HCT 116 cells by both RNA-Seq and qPCR analyses (Table 1, Figure 5C and D), we chose it for further study. After knocking out LSD1, both Western blot analysis of cellular extracts (Figure 6A) and ELISA analysis of secretion in culture media (Figure 6B) showed DKK1 increased. And we found that β-catenin, the key protein of the DKK1-related Wnt/β-catenin signaling pathway, decreased, while phosphorylated-β-catenin (p-β-catenin) increased significantly (Figure 6A). Additionally, the amount of free β-catenin translocated to the nucleus was decreased (Figure 6C), and the transcription of the signaling pathway target gene, c-Myc, was down-regulated (Figure 6A). The increase of β-catenin in the nucleus is indicative of the activation of the Wnt/β-catenin signaling pathway; thus, the results indicated that in WT HCT 116 cells, LSD1 activated the Wnt/β-catenin signaling pathway by down-regulating the pathway antagonist, DKK1.

## Discussion

In the detection of clinical samples by Western blot analysis, we found that LSD1 was highly expressed in tumor colorectal tissues in comparison to adjacent normal colorectal tissues (Figure 1), which suggested that LSD1 might function in colorectal tumorigenesis. To explore the role of LSD1 in colorectal tumorigenesis, we used somatic cell gene targeting to generate an LSD1 KO HCT 116 human colorectal cancer cell line as a research model for colorectal cancer (Figure 2). Gene targeting is one of the advanced research techniques used to explore gene function and the regulatory role of the signaling pathway. Compared with animal gene KO, somatic cell gene KO is much easier, less costly and more rapid. In addition, this technique overcomes the shortcomings of animal embryonic lethality. In contrast to the RNAi knockdown technique, somatic cell gene KO can completely delete the target gene. When compared to over-expression technique, somatic cell gene KO can avoid the influence of unstable artificial factors from over-expression, which makes the results more credible.

In this study, compared to WT colorectal cancer cells, LSD1 KO cells were less tumorigenic both in vivo and in vitro (Figure 3), which indicates that LSD1 plays a critical role in colorectal tumorigenesis. This finding was similar to that of Jin et al. [40]. Additionally, we found that the relationship between WT and LSD1 KO colorectal cancer cell lines was in good agreement with the relationship between tumor and adjacent normal colorectal tissues, which indicates that the LSD1 KO cell line is a good model for colorectal cancer research.

LSD1 is a demethylase, and it catalyzes the specific demethylation of H3K4me1/2 or H3K9me1/2; thus, before our experiments, it was reasonable to speculate that, after knocking out LSD1, the global methylation levels of H3K4me1/2 or H3K9me1/2 would increase. Surprisingly, in the experiment, these global methylation levels did not change significantly (Figure 5A). The result suggested that some unknown mechanism may compensate for the absent demethylase catalytic activity of LSD1. The result is similar to that of Wang et al., who performed in embryonic stem cells [12].

To explore the molecular basis of LSD1 and tumorigenesis, we analyzed the differential expression of the cells before and after knocking out LSD1 by RNA-Seq and found 2,663 differentially expressed genes (Figure 5B). Gene expression data are described in detail in Table S1. Statistical analysis allowed us to screen out 28 genes that exhibited highly significant differences (Q <0.01). Of these, 9 were down-regulated after knocking out LSD1, and the remaining 19 were up-regulated (Table 1 and Figure 5C). Interestingly, among these genes, 4 genes, including ADM [41], DKK1 [42], HAS3 [43] and SMURF2 [44], have been reported to be involved in colorectal tumorigenesis. Thus, we tried to find clues from these four for exploring the role of LSD1 in colorectal tumorigenesis. We took them for further confirmation by qPCR analysis and the results of this analysis showed that the differences in the expression of ADM, DKK1 and HAS3 were consistent with those measured using the RNA-Seq data (Table 1, Figure 5C and D). Because DKK1 was the gene with the most significant differential expression before and after knocking out LSD1 in HCT 116 cells, as shown by both RNA-Seq and qPCR analyses, and because it is an antagonist of the Wnt/β-catenin signaling pathway which plays an important role in development and tumorigenesis [30], [31], [45], we chose it for further research. Although the rest of the 2,663 differential expression genes were not analyzed further in this work, these genes do provide important reference values and clues for researchers in exploring the biological function of LSD1 and the role of LSD1 in colorectal tumorigenesis. The differential expression phenomenon observed in this work was similar to that observed by Huang et al. in their drug research. They reported that the inhibition of LSD1 in human colon carcinoma cells by biguanide and bisguanidine polyamine analogues resulted in a re-expression of aberrantly silenced genes, including SFRP1, SFRP4, SFRP5, and GATA5 [28].

The Wnt/β-catenin pathway in mammalian cells is activated when the Wnt ligand, a secreted factor, binds to cell-surface receptors of the Frizzled family and the LRP 5/6 co-receptor. In turn, this binding leads to the inhibition of a complex comprising APC, Axin, and GSK-3β. This complex normally plays a role in the phosphorylation and degradation of β-catenin by the ubiquitin/proteosome pathway [46]. When this complex is inhibited, β-catenin accumulates and translocates to the nucleus [46], where β-catenin binds to TCF/LEF factors and up-regulates the transcription of many cancer-related genes, including c-Myc [47], [48]. Constitutively active Wnt signaling is causally involved in the genesis of various cancers, including colorectal cancer [30], [31]. DKK1, a Wnt antagonist, blocks the Wnt/β-catenin pathway through competitive binding to the Wnt co-receptor LRP 5/6 and the transmembrane protein Kremen1/2 [49], [50]. DKK1 mediates tumor suppression in some cancer cells by blocking the Wnt/β-catenin pathway. Expression of DKK1 in these cells is associated with increased phosphorylation and degradation of β-catenin and down-regulation of c-Myc etc. oncogenes, so inhibiting cell growth [51], [52], [53]. In colorectal cancer, decreased DKK1 expression is observed [54], [55]. DKK1 has strong antiproliferative effects in colorectal cancer cell lines [54], [56] and dramatically reduces tumor burden in mouse xenografts [54]. Qi et al. reported that overexpression of DKK1 decreased the expression and intracellular distribution of β-catenin as well as the expression of c-Myc. Their study confirmed that DKK1 has inhibitory effects on colon cancer progression [42]. Overall, DKK1 is thought to act as a tumor suppressor gene. Inactivation or down-regulation of DKK1 can lead to various cancers, including colorectal cancer [54], [57], [58]. In our studies, we detected the key protein of the Wnt/β-catenin signaling pathway. The results showed that, after knocking out LSD1, secreted DKK1 increased, β-catenin decreased, p-β-catenin increased, and c-Myc decreased (Figure 6A and B), which suggested the decline of Wnt/β-catenin signaling pathway activation. The translocation of β-catenin to the nucleus is indicative of the activation of the Wnt/β-catenin signaling pathway. Thus, we further analyzed the protein levels of β-catenin in the cytoplasm and nucleus. We found that β-catenin was reduced in the cytoplasm and significantly reduced in the nucleus (Figure 6C). Because β-catenin was significantly reduced in the nucleus, it should also be significantly reduced in the cytoplasm. However, the experimental results showed that this protein was reduced in the cytoplasm but not as obviously as in the nucleus. We speculated that the free β-catenin in the cytoplasm is, in fact, significantly reduced but that the non-free β-catenin, which binds with E-cadherin on the inner side of the cell membrane in the cytoplasm, was also extracted due to the β-catenin/E-cadherin complexes were destroyed in the lysate during the protein extraction process. However, there was much more bound β-catenin than free β-catenin; therefore, the reduction was not as obvious as in the nucleus. Nevertheless, the experimental results still indicated that the amount of β-catenin translocated to the nucleus was significantly reduced and the obvious decline of Wnt/β-catenin signaling pathway activation.

DKK1 plays a key role here to link LSD1 to Wnt/β-catenin pathway and then to colorectal tumorigenesis. As LSD1 is a demethylase, which represses the transcription of target genes via demethylation of H3K4 by interacting with a variety of molecular partners [7], [8], and LSD1 may also repress the transcription of target genes via DNA CpG dinucleotide methylation by demethylating and stabilizing Dnmt1 [12], [40], we speculate that above might be two possible mechanisms of down-regulation of DKK1 by LSD1. As to the latter mechanism which involves Dnmt1, Wang et al. reported that LSD1 demethylated and stabilized Dnmt1 in embryonic stem cells [12], while Jin et al. reported that LSD1 could not perform the similar function in HCT 116 [40]. So we suggest that the former mechanism which involves H3K4 might be more possible; nevertheless, the exact mechanism needs further study.

**Figure 7:**
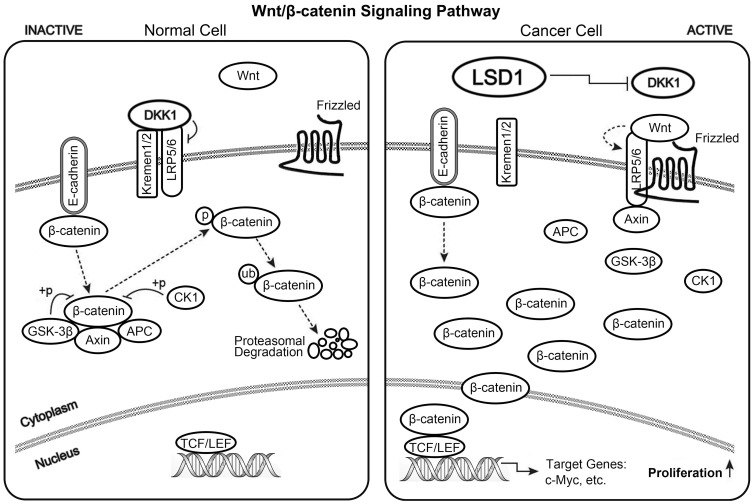
A diagram of the Wnt/β-catenin signaling pathway. We propose that LSD1 down-regulates the Wnt/β-catenin signaling pathway antagonist, DKK1, and that this down-regulation causes free β-catenin to avoid degradation and accumulate in the cytoplasm. The free β-catenin then translocated to the nucleus, activates the transcription of the signaling pathway target gene c-Myc, and finally leads to the aberrant proliferation of cells and tumorigenesis.

In conclusion, based on the existing reports and our studies, it is reasonable to speculate that LSD1 contributes to colorectal tumorigenesis via activation of the Wnt/β-catenin signaling pathway by down-regulating the pathway antagonist DKK1. Specifically, LSD1 down-regulates DKK1, which allows free β-catenin to avoid phosphorylation and degradation. As a result, free β-catenin then accumulates and translocates to the nucleus, where it up-regulates the transcription of downstream target genes, including the oncogene c-Myc; the overexpression of c-Myc then causes the cells to transition to a malignant phenotype. To illustrate this mechanism, we have included a simple diagram (Figure 7). To the best of our knowledge, this is the first report that links LSD1 and DKK1 and elucidates the impact of LSD1 on the Wnt/β-catenin signaling pathway activation and the pathway target genes to reveal the role of LSD1 in colorectal tumorigenesis.

## Supporting Information

Table S1 **RNA-Seq differential gene expression analysis of the cells before and after knocking out LSD1.**
(XLS)
