# Correction: Lysine-Specific Demethylase 1 (LSD1/KDM1A) Contributes to Colorectal Tumorigenesis via Activation of the Wnt/β-Catenin Pathway by Down-Regulating Dickkopf-1 (DKK1)

**DOI:** 10.1371/annotation/d090733e-1f34-43c5-a06a-255456946303

**Published:** 2013-12-10

**Authors:** Zebin Huang, Shangze Li, Wei Song, Xin Li, Qinshan Li, Zeyan Zhang, Yongqing Han, Xiaodong Zhang, Shiying Miao, Runlei Du, Linfang Wang

The title of the article is incorrect. The correct title is: "Lysine-Specific Demethylase 1 (LSD1/KDM1A) Contributes to Colorectal Tumorigenesis via Activation of the Wnt/β-Catenin Pathway by Down-Regulating Dickkopf-1 (DKK1)". The correct Citation is: Huang Z, Li S, Song W, Li X, Li Q, et al. (2013) Lysine-Specific Demethylase 1 (LSD1/KDM1A) Contributes to Colorectal Tumorigenesis via Activation of the Wnt/β-Catenin Pathway by Down-Regulating Dickkopf-1 (DKK1). PLoS ONE 8(7): e70077. doi:10.1371/journal.pone.0070077 

